# Infectiological Outcome of Total Hip and Total Knee Arthroplasty in Post-Traumatic and Primary Osteoarthritis

**DOI:** 10.3390/antibiotics13121186

**Published:** 2024-12-05

**Authors:** Maximilian Gresch, Nikolaus von Dercks, Nadine Dietze-Jergus, Andreas Roth, Christina Pempe

**Affiliations:** 1 Department of Orthopedics, Trauma and Plastic Surgery, University Hospital Leipzig, 04317 Leipzig, Germany; andreas.roth@medizin.uni-leipzig.de (A.R.);; 2 Department of Medical Management, University Hospital Leipzig, 04317 Leipzig, Germany; 3 Institute of Medical Microbiology and Virology, Department of Microbiology, University Hospital Leipzig, 04317 Leipzig, Germany

**Keywords:** periprosthetic joint infection, total hip arthroplasty, total knee arthroplasty, primary osteoarthritis, post-traumatic osteoarthritis, cefuroxime, clindamycin

## Abstract

**Background**: The objective of this study was to compare infection rates, pathogen species detection and antimicrobial susceptibility testing in patients with total hip arthroplasty (THA) and total knee arthroplasty (TKA) following post-traumatic osteoarthritis (PTOA) and primary osteoarthritis (POA). **Results**: Patients undergoing both THA and TKA were significantly more likely to have a PJI after PTOA than after POA (THA: 2.5% vs. 10.2%, *p* = 0.003; TKA: 3.2% vs. 10.3%, *p* = 0.028). The most frequently detected pathogen in both THA and TKA was *Staphylococcus* spp. Among patients with a PJI in THA, *Staphylococcus* spp. was detected in 47% after POA and 60% after PTOA. Among patients with a PJI in TKA, *Staphylococcus* spp. was isolated in 59% after POA and 80% after PTOA. The remaining pathogens were mainly *Enterococcus* spp., Enterobacterales and anaerobic bacteria. After THA, beta-lactam-resistant staphylococcal isolates were detected more frequently in PTOA patients than in POA patients (13% vs. 100%, *p* = 0.024). There was no difference in the beta-lactam staphylococcal resistance rate in patients after TKA (20% vs. 25%, *p* = 0.945). Furthermore, an analysis of susceptibility testing from all groups showed that significantly more pathogens were susceptible to vancomycin than to cefuroxime (76% vs. 45%, *p* < 0.001) or clindamycin (76% vs. 52%, *p* = 0.007). **Methods**: A retrospective analysis was performed using clinic-owned data during the period January 2016–December 2020. A total of 1485 patients following primary implantation of THA or TKA due to PTOA or POA were included. Early-onset periprosthetic joint infection (PJI), defined according to the 2018 Definition of Periprosthetic Hip and Knee Infection Criteria, was evaluated. **Conclusions**: Therefore, the use of vancomycin as a perioperative prophylaxis should be discussed under benefit/risk consideration in further studies.

## 1. Introduction

THA and TKA are commonly performed and well-established surgical procedures for the treatment of hip and knee osteoarthritis (OA). Both procedures have high success rates, with the majority of patients reporting significant improvements in pain, function and mobility. These outcomes lead to an enhanced quality of life for most patients after surgery [1,2,3]. However, the results of THA and TKA can vary depending on the etiology of the OA. PTOA patients may be at an elevated risk for postoperative complications, including PJI, in comparison to POA patients [4]. This could be attributed to the presence of disparate comorbidities, risk factors and a distinct pathophysiology that results in complex joint degenerations [5,6,7].

PJI is one of the most significant complications, which can lead to severe morbidity and loss of function of the affected joint. This necessitates revision surgery and prolonged hospitalization, which in turn lead to high socioeconomic costs [3,8,9,10]. Therefore, effective prevention strategies are essential, including the use of perioperative antibiotic prophylaxis to reduce the risk of PJI following arthroplasty [3,11,12,13]. Despite recent improvements in antibiotic regimens that have contributed to better outcomes in infection prevention, and although the incidence of PJI after POA is relatively low at up to 2%, infection rates after PTOA, at up to 10%, still appear to be considerably higher [3,4,8,10,12,14,15,16]. It is for this reason that further research is required in this field, with the objective of reducing the incidence of PJI following PTOA. Cefuroxime and, in cases of penicillin allergy, clindamycin are commonly used for perioperative antibiotic prophylaxis across a range of patient populations for the prevention of surgical site infections [17,18]. Given the elevated incidence of PJI following PTOA, it remains unclear whether conventional antibiotic regimens are sufficient for the prevention of PJI or whether alternative antibiotics are necessary.

The aim of this study was to compare periprosthetic joint infection rates in patients with total hip and knee arthroplasty after POA and PTOA, to detect the most responsible pathogen strains in both groups, and to compare the microbiological findings using cefuroxime and clindamycin as perioperative antibiotic prophylaxis.

## 2. Results

A total of 1485 patients were included in this study. Of these, 948 (63.8%) underwent THA [POA: 685 (72.3%); PTOA: 68 (7.2%)], while 537 (36.2%) underwent TKA [POA: 437 (81.4%); PTOA: 45 (8.4%)]. A total of 1395 patients (93.9%) received cefuroxime and 90 (6.1%) received clindamycin as perioperative antibiotic prophylaxis. The median follow-up was 541 days (IQR 179-1045 days). A total of 361 patients (24.3%) were lost to follow-up. PJI was diagnosed in 46 patients (3.1%) [THA: 14 POA, 5 PTOA and 7 other OA of the hip (dysplasia, femoral head necrosis); TKA: 13 POA, 4 PTOA and 3 other OA of the knee (rheumatoid arthritis)]. Due to the small number of cases of PJI after coxarthrosis and gonarthrosis that were not due to POA or PTOA, and the fact that these represent separate subgroups with an associated risk profile, separate group comparisons were not carried out for patient characteristics and pathogen species. However, these cases were taken into account in the overall assessment of pathogen resistance rates in order to obtain an overview of all cases.

### 2.1. Characteristics of Patients with PJI After THA and TKA

A total of 41.3% of all patients with PJI were female, and the mean age was 65 (IQR 60–74) years. The median BMI was 29.7 (27–35.5) kg/m^2^. The median duration of surgery was 83 (60–103) minutes. Patients with PTOA of the hip and knee were observed to be significantly younger (*p* = 0.002, *p* = 0.009) and the hip arthroplasty procedure was found to take significantly longer (*p* = 0.003). There was no difference between the two groups regarding gender, BMI, diabetes mellitus or ASA classification (see Table 1 and Table 2). After THA, patients with PTOA significantly more often experienced PJI than patients with POA (10.2% vs. 2.5%, *p* = 0.003). We found the same pattern after TKA (10.3% vs. 3.2%, *p* = 0.028).

A total of 58 pathogens (see Table 3, Table 4, Table 5 and Table 6) were isolated: only one bacterial species in 35 patients, two in 8 patients, and three or four in 1 patient each.

### 2.2. Analysis of Pathogens and Antibiotic Susceptibility

#### 2.2.1. Total Hip Arthroplasty

Among patients with PJI in THA after POA, 13 of the 17 (76%) isolated pathogens were Gram-positive (*Staphylococcus* spp. (8 of 17; 47%), *Streptococcus* spp., *Enterococcus* spp. and anaerobic bacteria), and 4 of the 17 (24%) were Gram-negative (Enterobacterales) (Table 3). Among the *Staphylococcus* spp. cases, three strains belonged to coagulase-negative Staphylococci (CoNS), and five strains were identified as *S. aureus*. Susceptibility testing of the isolated strains showed that 10 out of 17 (59%) strains were resistant to the perioperative antibiotic prophylaxis administered. Among them, one CoNS, one *Streptococcus* spp., and eight strains (47%) with intrinsic resistance to either cefuroxime or clindamycin were observed.

Among patients with PJI in THA after PTOA, four of the five (80%) isolated pathogens were Gram-positive, including three CoNS strains, and one of five (20%) were Gram-negative. All strains tested were resistant to perioperative antibiotic prophylaxis (Table 4). Comparing the resistance rate of all pathogens in POA versus PTOA of the hip (in relation to cefuroxime and clindamycin), there was no significant difference (*p* = 0.189). Yet, in the group of patients after PTOA, the detected CoNS had a significantly higher likelihood of resistance than after POA (*p* = 0.024), where susceptible *S. aureus* strains were mainly found.

If cefuroxime was administered perioperatively, more isolates susceptible to cefuroxime were detected in vitro in the POA group. Considering the susceptibility of these isolates to clindamycin, all strains, except one anaerobic isolate, were also susceptible to clindamycin. However, there was no difference in patients after PTOA. Comparing the resistance against cefuroxime of potentially susceptible pathogens in both groups, there was no difference (*p* = 0.296).

#### 2.2.2. Total Knee Arthroplasty

Among patients with PJI in TKA after POA, 16 of the 17 (94%) isolated pathogens were Gram-positive (*Staphylococcus* spp. (10 of 17; 59%), *Enterococcus* spp., *Bacillus* spp., *Corynebacterium* spp. and *Micrococcus* spp.) and 1 of the 17 (6%) was Gram-negative (Enterobacterales) (Table 5). As shown for THA, *S. aureus* (6 of 10) was mainly detected among the *Staphylococcus* spp. cases. Regarding the detected strains, 8 of 17 (47%) were resistant to the perioperative antibiotic prophylaxis, including 5 cases (29%) of intrinsic resistance. For *Staphylococcus* spp., 2 of 10 (20%) were resistant to the antibiotic prophylaxis given. Among patients with PJI in TKA after PTOA, all the isolated pathogens were Gram-positive, and two of five (40%) were resistant to the perioperative antibiotic prophylaxis. Four of five (80%) were *Staphylococcus* spp., of which one (CoNS) (25%) was resistant (Table 6). 

Comparing the resistance rate of all pathogens in POA versus PTOA of the knee, there was no significant difference (*p* = 0.82), nor was there a difference when comparing staphylococcal resistance. Evaluating the efficacy of cefuroxime and clindamycin on potentially susceptible pathogens, there was no difference in either group (*p* = 0.333).

### 2.3. Alternatives for Perioperative Antibiotic Prophylaxis

Of the 1395 patients who received cefuroxime, 30 (2.2%) developed a PJI. In comparison, 6 of the 90 (6.7%) patients who received clindamycin as perioperative antibiotic prophylaxis also developed this complication (*p* = 0.019), representing a significantly higher incidence than that observed in the cefuroxime group.

As the aim of perioperative antibiotic prophylaxis is to reduce pathogens of the resident skin flora, such as *Staphylococcus* spp., these pathogens were evaluated separately. Considering the efficacy of cefuroxime and clindamycin in patients receiving these agents as prophylaxis against *Staphylococcus* spp., 6 of 25 (24%) were resistant to cefuroxime and 100% (5 of 5) were resistant to clindamycin (*p* = 0.006).

Microbiological testing and evaluation of all 58 isolated strains showed that 76% of all pathogens were susceptible to vancomycin, 45% to cefuroxime (44 vs. 26, *p* < 0.001) and 52% to clindamycin (44 vs. 30, *p* = 0.007) (Table 7).

In the group of patients after POA and total knee arthroplasty, 8 of the 17 (47%) isolated pathogens were resistant to cefuroxime and 1 of the 17 (6%) were resistant to vancomycin, which was an intrinsic resistance of *K. oxytoca* (*p* = 0.041). The other groups showed no statistically significant differences.

## 3. Discussion

The aim of this study was to compare patients with total hip arthroplasty and total knee arthroplasty after PTOA and POA with regard to infection rates, detected strains and their antibiotic susceptibility patterns. Differences in infection rates were statistically proven, pathogens and their antibiotic susceptibility were identified, and alternatives to the current perioperative prophylaxis were determined.

### 3.1. Infection Rates

Reported infection rates vary but are generally low, ranging from 0.5% to 2% after primary arthroplasty [3,8,10,12,14,16]. Infection rates after PTOA were described to be much higher, up to 10% [3,4,10,15].

Certain patient groups are at higher risk for PJI, especially patients with comorbidities such as obesity, diabetes, coronary artery disease, nicotine abuse or rheumatoid arthritis [10,13,14,16,19]. Our results show that patients with either THA or TKA had a significantly higher incidence of PJI after PTOA than after POA, confirming the findings in the literature (THA: 10.2% vs. 2.5%, *p* = 0.003; TKA: 10.3% vs. 3.2%, *p* = 0.028). This could be due to prolonged surgery time, abnormal soft tissue, undetected bacterial colonization or osteosynthetic material in situ that is less accessible to the immune system and microvascular circulation and that favors the formation of bacterial biofilms [3,10,11,20]. Morison et al. found that 7% of patients with THA developed PJI after PTOA and identified multiple previous surgeries and retained hardware from previous acetabular reconstructions as risk factors [4]. Hemmann et al. isolated pathogens, primarily *Staphylococcus* spp., in 10% of patients with THA and previous internal fixation of the proximal femur; they found that prolonged operation time, scar tissue from the hip approach, and synovitis may contribute to an increased risk of infection [15]. In our study, patients after PTOA also had a longer duration of surgery than patients after POA, which could have an influence on the higher rates of PJI. The younger age of patients in our cohort who underwent either THA or TKA may be attributed to the earlier onset of symptoms following PTOA compared to POA (*p* = 0.002, *p* = 0.009). These findings are consistent with those reported in the literature [7].

### 3.2. Pathogen Strains

The most common pathogens causing PJI are *S. aureus* (including MRSA), coagulase-negative staphylococci, *Streptococcus* spp., and *Enterococcus* spp. [3,10,11,12,13,15,19,20]. In accordance with this, *Staphylococcus* spp. was also predominantly identified in this study. In the group of patients following THA, *Staphylococcus* spp. was detected in 47% after POA and 60% after PTOA (*p* = 0.704). In the group of patients with THA after PTOA, the detected *Staphylococcus* spp. had a significantly higher likelihood of resistance than after POA (*p* = 0.024). However, as only three isolates could be examined in this group, further investigations are necessary.

In patients with POA vs. PTOA TKA, *Staphylococcus* spp. was isolated in 59% and 80% of cases, respectively (*p* = 0.493). There was no significant difference in the spectrum of pathogens between POA and PTOA in THA and TKA patients.

### 3.3. Perioperative Antibiotic Prophylaxis

Cefuroxime-resistant pathogens were detected in 49% of patients with PJI who received cefuroxime as perioperative prophylaxis. In the group of patients who received clindamycin as perioperative prophylaxis, all pathogens detected were assessed as resistant in susceptibility testing, with resistance to cefuroxime also present in five out of seven isolates.

Despite perioperative cefuroxime prophylaxis, isolates were discovered that were susceptible to cefuroxime. This could be due to an overly rapid drop in the effective level of cefuroxime or insufficient accumulation in the target area. To counteract this, the use of higher single doses or the repeated administration of perioperative prophylaxis, e.g., a follow-up dose for surgery procedures taking longer than 3 h, should be considered.

Using clindamycin for perioperative prophylaxis, only clindamycin-resistant isolates were detected. This could indicate better efficacy of the substance due to better accumulation in the target area.

Various alternatives for perioperative prophylaxis were determined based on the evaluation of susceptibility testing of the isolated pathogens and references from microbiology. In our study, we found that the likelihood of pathogen susceptibility was significantly higher with vancomycin compared with cefuroxime (76% vs. 45%) or clindamycin (76% vs. 52%).

Bosco et al. stated that vancomycin is generally recommended for patients with a beta-lactam allergy or with a high risk for MRSA colonialization or infection [13]. Courtney et al. reported that routine addition of vancomycin to cefazolin prophylaxis in all patients undergoing primary hip and knee arthroplasty is associated with a higher risk of acute kidney injury [21], whereas Burger et al. found that the addition of vancomycin to cefazolin reduced PJI rates in primary hip and knee arthroplasty with a low risk of renal impairment [22]. Tan et al. showed that patients who received vancomycin alone demonstrated reduced odds of Gram-positive organisms and methicillin-resistant *Staphylococcus aureus* [23]. Since the literature defines acute kidney injury due to vancomycin-induced nephrotoxicity as at least two or three consecutive high serum creatinine concentrations after several days of therapy, the risk after administration of a single-shot dose can be considered low [24]. Older age, longer treatment courses and higher trough serum vancomycin concentrations were identified as risk factors for vancomycin-induced nephrotoxicity [25]. 

In summary, in addition to clindamycin, vancomycin should be considered as an alternative to cefuroxime in patients with a penicillin allergy. Furthermore, the use of vancomycin as perioperative prophylaxis in patients at high risk for developing PJI, such as patients with PTOA of the hip and knee, should be discussed with the consideration of patient characteristics such as renal function.

### 3.4. Limitations

This study has limitations mainly related to the retrospective study design. First, patient characteristics were analyzed only from patients with known or suspected surgical site infections and not from patients without infections. Second, the total number of patients analyzed was small because of the relatively low incidence of PJI even over a 5-year period. Third, infections also occurred in patients with an isolated pathogen that was susceptible to perioperative antibiotic prophylaxis, so it is unclear whether a reduction in antibiotic resistance would lead to a proportional decrease in infections. Fourth, all patients received cefuroxime or clindamycin as perioperative prophylaxis; other antibiotics were not administered and can therefore only be assessed by comparison of antibiograms.

## 4. Materials and Methods

### 4.1. Patient Selection

To determine infectiological outcomes, a retrospective study of all patients older than 18 years who underwent primary hip or knee arthroplasty at Leipzig University Hospital between 1 January 2016 and 31 December 2020 was performed. Patients under 18 years of age, with previous joint surgery within the last 90 days, with healed joint infection, with (pathological) fracture or with failed osteosynthesis were excluded. The patient data were prepared by the Data Integration Center of Leipzig University Hospital. The data were accessed between 1 January 2021 and 31 December 2021.

### 4.2. Procedure

All patients received perioperative antibiotic prophylaxis with cefuroxime (1500 mg) as clinic internal standard treatment or, if allergic to cephalosporins, clindamycin (600 mg) as a single dose 30–60 min prior to skin incision. No further antibiotics were administered for prophylaxis. Repeated administration of perioperative prophylaxis was carried out for surgical procedures lasting longer than 3 h. Higher single doses of 3000 mg cefuroxime were administered if the patient weighed more than 120 kg. Surgery was performed by senior physicians specialized in arthroplasty [11,13]. The standard treatment for hip OA was arthroplasty using the same uncemented prosthesis model with polyethylene ceramic glide components in all patients. The use of cemented prostheses and metallic heads was only employed in exceptional cases. The standard treatment for knee OA was arthroplasty using the same cemented prothesis model in all patients.

### 4.3. Evaluation

All patients with PJI within the first 45 days after surgery according to the 2018 Definition of Periprosthetic Hip and Knee Infection Criteria were evaluated, considering two positive cultures or the presence of a sinus tract as major criteria and diagnosis of PJI among other minor criteria [26,27,28]. Based on our hypothesis that during primary prosthesis implantation in patients with PTOA and foreign material in place, the material was already bacterially colonized, samples were taken and examined microbiologically. The classification of pathogens as “Susceptible, standard dosing regimen” (S), “Susceptible, increased exposure” (I), and “Resistant” (R) was based on “The European Committee on Antimicrobial Susceptibility Testing. Breakpoint tables for interpretation of MICs. Version 13.0, 2023”. Only pathogens classified as “Susceptible, standard dosing regimen” (S) were interpreted as susceptible to the antibiotic prophylaxis given. Pathogens classified as “Susceptible, increased exposure” (I) were interpreted as resistant as it is unlikely that a single-shot dose would reach the required elevated concentration in the target tissue. Pathogens with the note “not tested/unsuitable for treatment” and with the interpretation “no evaluation” were also classified as resistant, as they exhibit intrinsic resistance to the respective antibiotic prophylaxis.

### 4.4. Statistics

SPSS version 29 software (SPSS Inc., Chicago, IL, USA) was used for statistical analyses, and a Shapiro–Wilk test was performed to determine the distribution of all variables. For continuous data, if a normal distribution of the variables could be assumed, a *t*-test was used; otherwise, a Mann–Whitney U test was performed. For categorical data, the exact Fisher test was used. A multivariant analysis was conducted on patient characteristics for the purpose of statistical data interpretation. The significance level was set at *p* < 0.05. Data are presented as the median with the interquartile range (IQR) in parentheses. 

## 5. Conclusions

Patients with THA or TKA following PTOA were more likely to develop PJI than patients with POA. In our patient population, there was an increased rate of resistant *Staphylococcus* spp. in THA patients with PTOA versus POA. An evaluation of susceptibility testing from all groups showed that most pathogens were Gram-positive and that more pathogens were susceptible to vancomycin than to cefuroxime or clindamycin. Using cefuroxime, the administration of higher single doses or the repeated administration of cefuroxime should be considered. The use of vancomycin for perioperative prophylaxis in patients at increased risk of PJI, e.g., after PTOA, should be discussed in the context of further studies, weighing the risks and benefits.

## Figures and Tables

**Table 1:** Characteristics of patients with PJI after total hip arthroplasty (THA). POA: primary osteoarthritis; PTOA: post-traumatic osteoarthritis; IQR: interquartile range; BMI: Body Mass Index; ASA: American Society of Anesthesiologists grade; PJI: periprosthetic joint infection.

THA		POA	PTOA	*p*-Value
Female gender	n (%)	5 (35.7)	3 (60)	0.603
Male gender	n (%)	9 (64.3)	2 (40)	
Age (years)	Median (IQR)	76 (67–80)	58 (52–65)	0.002
BMI (kg/m^2^)	Median (IQR)	28 (26–37)	38 (27–40)	0.336
Diabetes mellitus	n (%)	3 (21.4)	1 (20)	1.000
ASA				0.603
I	n (%)	0 (0)	0 (0)	
II	n (%)	5 (35.7)	3 (60)	
III	n (%)	9 (64.3)	2 (40)	
IV	n (%)	0 (0)	0 (0)	
V	n (%)	0 (0)	0 (0)	
Duration of surgery (min)	Median (IQR)	58 (51–83)	133 (83–158)	0.002
Perioperative antibiotic prophylaxis				
Cefuroxime	n (%)	11 (78.6)	4 (80)	1.000
Clindamycin	n (%)	3 (21.4)	1 (20)	
Time of single shot application before skin incision (min)	Median (IQR)	35 (29–43)	40 (28–46)	0.486

**Table 2:** Characteristics of patients with PJI after total knee arthroplasty (TKA). POA: primary osteoarthritis; PTOA: post-traumatic osteoarthritis; IQR: interquartile range; BMI: Body Mass Index; ASA: American Society of Anesthesiologists grade; PJI: periprosthetic joint infection.

TKA		POA	PTOA	*p*-Value
Female gender	n (%)	6 (46.2)	1 (25)	0.603
Male gender	n (%)	7 (53.8)	3 (75)	
Age (years)	Median (IQR)	68 (61–76)	54 (38–64)	0.009
BMI (kg/m^2^)	Median (IQR)	30 (27–33)	36 (29–37)	0.164
Diabetes mellitus	n (%)	2 (15.4)	1 (25)	1.000
ASA				0.584
I	n (%)	0 (0)	0 (0)	
II	n (%)	9 (69.2)	2 (50)	
III	n (%)	4 (30.8)	2 (50)	
IV	n (%)	0 (0)	0 (0)	
V	n (%)	0 (0)	0 (0)	
Duration of surgery (min)	Median (IQR)	80 (68–103)	134 (99–275)	0.116
Perioperative antibiotic prophylaxis				
Cefuroxime	n (%)	12 (92.3)	3 (75)	0.426
Clindamycin	n (%)	1 (7.7)	1 (25)	
Time of single shot application before skin incision (min)	Median (IQR)	28 (21–34)	36 (34–39)	0.491

**Table 3:** Minimum inhibitory concentration (MIC) of bacteria isolated from patients with PJI after THA in POA. S: susceptible, standard dosing regimen; I: susceptible, increased exposure; R: resistant; -: not tested/unsuitable for treatment; *: no evaluation.

MIC (mg/L)	Penicillin	Ampicillin/ Sulbactam	Cefuroxime	Clindamycin	Linezolid	Vancomycin
Perioperative antibiotic prophylaxis: cefuroxime						
*C. koseri*	-	>32 (R)	4 (I)	-	-	-
*E. cloacae*	-	32 (R)	8 (R)	-	-	-
*E. cloacae*	-	16 (R)	8 (R)	-	-	-
*E. faecalis*	-	2 (S)	>32 (*)	-	2 (S)	0.5 (S)
*E. faecalis*	-	0.5 (S)	>32 (*)	-	2 (S)	0.5 (S)
*F. magna*	0.0625 (S)	0.0625 (S)	-	>256 (R)	-	-
*F. magna*	0.0625 (S)	0.0625 (S)	-	0.5 (S)	-	-
*S. aureus*	<0.03125 (S)	<0.25 (S)	2 (S)	0.0625 (S)	0.5 (S)	0.5 (S)
*S. aureus*	1 (R)	0.5 (S)	1 (S)	0.125 (S)	1 (S)	1 (S)
*S. aureus*	0.0625 (S)	<0.25 (S)	2 (S)	0.0625 (S)	1 (S)	0.5 (S)
*S. aureus*	1 (R)	<0.25 (S)	1 (S)	0.0625 (S)	1 (S)	1 (S)
*S. aureus*	<0.03125 (S)	<0.25 (S)	2 (S)	0.125 (S)	2 (S)	1 (S)
*S. capitis*	<0.03125 (S)	<0.25 (S)	<0.25 (S)	0.0625 (S)	0.25 (S)	0.5 (S)
*S. epidermidis*	<0.03125 (S)	<0.25 (S)	0.5 (S)	0.125 (S)	1 (S)	1 (S)
Perioperative antibiotic prophylaxis: clindamycin						
*P. mirabilis*	-	1 (S)	0.5 (I)	-	-	-
*S. epidermidis*	4 (R)	2 (R)	4 (R)	>4 (R)	0.5 (S)	1 (S)
*S. salivarius*	0.25 (S)	0.125 (S)	0.5 (S)	>4 (R)	-	-

**Table 4:** Minimum inhibitory concentration (MIC) of bacteria isolated from patients with PJI after THA in PTOA. S: susceptible, standard dosing regimen; I: susceptible, increased exposure; R: resistant; -: not tested/unsuitable for treatment; *: no evaluation.

MIC (mg/L)	Penicillin	Ampicillin/ Sulbactam	Cefuroxime	Clindamycin	Linezolid	Vancomycin
Perioperative antibiotic prophylaxis: cefuroxime						
*K. pneumoniae*	-	-	2 (I)	-	-	-
*S. epidermidis*	4 (R)	2 (R)	8 (R)	4 (R)	0.5 (S)	2 (S)
*S. epidermidis*	>4 (R)	8 (R)	>32 (R)	>4 (R)	0.25 (S)	0.5 (S)
Perioperative antibiotic prophylaxis: clindamycin						
*C. aurimucosum*	4 (R)	-	2 (*)	>256 (R)	0.25 (S)	0.25 (S)
*S. epidermidis*	0.25 (R)	<0.25 (S)	0.5 (S)	>4 (R)	0.5 (S)	1 (S)

**Table 5:** Minimum inhibitory concentration (MIC) of bacteria isolated from patients with PJI after TKA in POA. S: susceptible, standard dosing regimen; I: susceptible, increased exposure; R: resistant; -: not tested/unsuitable for treatment; *: no evaluation.

MIC (mg/L)	Penicillin	Ampicillin/ Sulbactam	Cefuroxime	Clindamycin	Linezolid	Vancomycin
T						
*B. cereus*	>4 (R)	8 (R)	>32 (R)	0.5 (S)	0.5 (S)	1 (S)
*C. durum*	0.125 (S)	-	-	4 (R)	2 (S)	0.25 (S)
*E. faecalis*	2 (*)	2 (S)	>32 (*)	>4 (*)	2 (S)	1 (S)
*E. faecalis*	4 (*)	2 (S)	>32 (*)	>4 (*)	1 (S)	1 (S)
*E. faecalis*	2 (*)	1 (S)	>32 (*)	>4 (*)	1 (S)	1 (S)
*K. oxytoca*	-	16 (R)	2 (I)	-	-	-
*M. luteus*	<0.03125 (S)	<0.25 (S)	0.5 (S)	<0.03125 (S)	<0.125 (S)	<0.125 (S)
*S. aureus*	0.0625 (S)	<0.25 (S)	1 (S)	0.125 (S)	1 (S)	1 (S)
*S. aureus*	0.0625 (S)	<0.25 (S)	1 (S)	0.125 (S)	1 (S)	1 (S)
*S. aureus*	>4 (R)	<0.25 (S)	1 (S)	0.125 (S)	1 (S)	0.5 (S)
*S. aureus*	1 (R)	0.5 (S)	2 (S)	0.125 (S)	1 (S)	0.5 (S)
*S. aureus*	>4 (R)	1 (S)	2 (S)	0.125 (S)	2 (S)	1 (S)
*S. aureus*	0.5 (R)	0.5 (S)	0.5 (S)	<0.03125 (S)	0.25 (S)	0.5 (S)
*S. epidermidis*	2 (R)	2 (R)	8 (R)	0.5 (R)	1 (S)	2 (S)
*S. epidermidis*	>0.5 (R)	(S)	(S)	<0.25 (S)	(S)	<0.5 (S)
*S. hominis*	<0.03125 (S)	<0.25 (S)	0.5 (S)	<0.03125 (S)	0.25 (S)	1 (S)
Perioperative antibiotic prophylaxis: clindamycin						
*S. epidermidis*	>4 (R)	4 (R)	>32 (R)	>4 (R)	0.5 (S)	2 (S)

**Table 6:** Minimum inhibitory concentration (MIC) of bacteria isolated from patients with PJI after TKA in PTOA. S: susceptible, standard dosing regimen; R: resistant; *: no evaluation.

MIC (mg/L)	Penicillin	Ampicillin/ Sulbactam	Cefuroxime	Clindamycin	Linezolid	Vancomycin
Perioperative antibiotic prophylaxis: cefuroxime						
*S. aureus*	<0.03125 (S)	<0.25 (S)	2 (S)	0.125 (S)	2 (S)	1 (S)
*S. carnosus*	<0.03125 (S)	<0.25 (S)	1 (S)	0.0625 (S)	1 (S)	0.5 (S)
*S. epidermidis*	>4 (R)	1 (S)	<0.25 (S)	0.0625 (S)	0.25 (S)	1 (S)
*S. epidermidis*	>4 (R)	32 (R)	>32 (R)	>4 (R)	0.25 (S)	0.5 (S)
Perioperative antibiotic prophylaxis: clindamycin						
*E. faecalis*	2 (*)	1 (S)	>32 (*)	>4 (*)	1 (S)	0.5 (S)

**Table 7:** Antibiotic susceptibility rates of all isolated pathogens, including primary, post-traumatic and other osteoarthritis. * including macrolide-lincosamide streptogramin B resistance.

Cefuroxime	n (%)	26 (45)
Clindamycin *	n (%)	30 (52)
Ampicillin/Sulbactam	n (%)	40 (69)
Linezolid	n (%)	44 (76)
Vancomycin	n (%)	44 (76)

## Data Availability

The original contributions presented in the study are included in the article; further inquiries can be directed to the corresponding authors.
